# Infliximab-graded challenge in a patient with Crohn’s disease and adalimumab hypersensitivity

**DOI:** 10.1186/1710-1492-8-S1-A21

**Published:** 2012-11-02

**Authors:** Christine H Song, Jaclyn A Quirt, Jason K Lee

**Affiliations:** 1 Division of Allergy and Clinical Immunology, St. Michael’s Hospital, University of Toronto, Toronto, Ontario, Canada, M5B 1W8; 2 Department of Internal Medicine, McMaster University, Hamilton, Ontario, Canada, L8N 3Z5

## Background

Infliximab and adalimumab are monoclonal antibodies to tumor necrosis factor alpha (TNF-α) used in the treatment of various inflammatory disorders. Infliximab, a chimeric monoclonal antibody, is postulated to be more immunogenic as it is not entirely humanized.

Despite reports of adalimumab treatment in patients after an adverse reaction to infliximab, there is a paucity of literature reporting the converse – treatment with infliximab after adverse reaction to adalimumab. Thus, it is difficult to estimate the risk of cross-sensitization. Graded drug challenges are utilized for patients unlikely to be allergic to a specific drug, but where concern for a reaction remains.

## Objective

To present a patient with Crohn’s disease and prior hypersensitivity to adalimumab who successfully underwent a graded intravenous challenge with infliximab.

## Methods

The patient previously had acute generalized urticaria due to adalimumab, with corresponding positive intradermal skin tests. Because her bowel disease activity was severe, her gastroenterologist preferred to start another anti-TNF agent. Infliximab was the chosen alternative. She underwent an infliximab-graded challenge in an outpatient clinic staffed by trained allergists.

## Results

The patient received infliximab during the graded challenge without adverse reactions.

## Conclusions

This is the first case, to our knowledge, to demonstrate an infliximab-graded challenge for a patient with a prior reaction to adalimumab. For patients requiring TNF-α inhibitors, but with previous reactions and concern for cross-sensitization, a graded challenge with the first dose of an alternate agent under observation by care providers trained to manage adverse drug reactions, may be a safe approach.

